# Neglected zoonotic diseases in Nigeria: role of the public health veterinarian

**DOI:** 10.11604/pamj.2019.32.36.15659

**Published:** 2019-01-18

**Authors:** Nusirat Elelu, Julius Olaniyi Aiyedun, Ibraheem Ghali Mohammed, Oladapo Oyedeji Oludairo, Ismail Ayoade Odetokun, Kaltume Mamman Mohammed, James Olaniyi Bale, Saka Nuru

**Affiliations:** 1 Department of Veterinary Public Health and Preventive Medicine, University of Ilorin, Kwara State, Nigeria

**Keywords:** Anthrax, brucellosis, rabies, bovine TB, cysticercosis, echinococcosis, trypanosomiasis, leishmaniasis

## Abstract

Zoonotic diseases accounts for about 75% of emerging infectious disease and can be devastating to both human and animal health globally. A subset of zoonotic diseases is referred to as “neglected zoonotic diseases - NZDs” as they mainly affect poor populations who live in close proximity to domestic or wild animals often in areas where access to health and adequate sanitary facilities are not available. Furthermore, underestimation of the burden of NZD has continually led to its further neglect in least developed countries such as Nigeria. Controlling zoonotic infections including NZDs in animals is crucial in reducing human infections. Veterinarians provides an understanding of the epidemiology of infectious diseases in animal population and are therefore integral for the overall reduction in global burden of NZDs worldwide. Due to the current lack of and in some cases weak involvement of Veterinarians in policy issues related to zoonotic diseases, there is need to elucidate their importance in NZDs control in Nigeria. This review therefore summarises the neglected zoonotic diseases so far documented in Nigeria and also highlight the important role of the Veterinarian in their prevention and control within both human and animal population. Important recommendations to strengthen the role of the public health Veterinarian for sustainable control of NZDs were made.

## Introduction

Zoonoses are diseases that are naturally transmissible between man and vertebrate animals [1]. About 75% of the new diseases that have affected humans over the past 10 years have been caused by pathogens originating from an animal or from products of animal origin [2]. About 20% of all human morbidity and mortality in the least developed countries of the world are attributable to endemic zoonoses [3-5]. With increasing ecological changes which favours development of some vectors of infection; urbanization which has led to increased contact with wildlife, which also constitute a significant reservoir of zoonotic infectious diseases as well as other socio-cultural behaviour such as farming, hunting and tourism further encourages the emergence and re-emergence of zoonotic infectious diseases [6]. A subset of zoonotic infectious diseases is referred to as “neglected zoonotic disease - NZDs”. These diseases are termed “neglected” as they mainly affect poor populations who live in close proximity to domestic or wild animals often in areas where health and adequate sanitary conditions are scarce [7]. Reports have shown that these diseases were further neglected due to the fact that their burden is usually concentrated in developing countries, where the majority of effort in recent years has focused on HIV/AIDs, tuberculosis, and malaria as well as ineffective diagnostic capacity and poor health care delivery systems that may result in underestimation of disease burden [8]. The WHO has identified eight diseases as NZD including: anthrax, bovine tuberculosis, brucellosis, cysticercosis, echinococcosis, leishmaniasis, rabies and human African trypanosomiasis [1]. Neglected zoonotic diseases have a dual burden because they can be devastating to both public health and animal health and the most vulnerable people are the millions of poor livestock keepers found globally [9]. Zoonotic disease exhibits clinical signs that mimics malaria, typhoid and HIV/AIDS in humans, leading to incorrect diagnoses and under-reporting hence underestimation of the disease burden [10, 11]. Neglected zoonotic diseases such as brucellosis constitutes an important public health problem throughout the world, particularly in the tropics, where its control is restricted by inadequate infrastructure, limited resources and lack of information on its significance and distribution [1]. Welburn *et al.* (2015), suggests that by targeting the animal reservoir to have optimal health, NZDs may reduce the risk of infection for humans, as well as ultimately leading to improved livelihoods through increased animal productivity [11]. In Nigeria, NZD are currently endemic in the animal population and has been reported across various parts of the country. Moreover, there has been an upsurge in the emergence of zoonotic infection in Nigeria, claiming several lives in recent times. The WHO has identified the need for collaborative effort to combat these important group of diseases. Hence the importance of the public health Veterinarian in their prevention and control cannot be overemphasized. This review would summarise the important zoonoses so far reported in Nigeria and also highlight the important role of the Veterinarian in prevention and control in both human and animal population.

## Methods

Literature search was conducted in the Google and PubMed biomedical databases including the each of the 8 NZDs: “Anthrax”, “Bovine tuberculosis”, “Brucellosis”, “Rabies”, “Cysticercosis”, “Taeiasis”, “Leishmaniasis” and “Human African Trypanosomiasis”; “Zoonoses” and “Nigeria” as keywords. We also searched through indexes of AJOL journals that published African research. Upon assessment of publication abstract, articles published in English and with relevant prevalence data on human NZDs from Nigeria were included. The bibliographies of the articles that turned up in the initial search were also used to find other references relevant to NZDs in Nigeria. Articles on animal cases and general information about neglected diseases were also included to provide background on the subject matter.

## Current status of knowledge

### Neglected Zoonoses of Importance in Nigeria

**Anthrax:** anthrax is an acute, often fatal re-emerging zoonotic infection caused by gram-positive spore-forming bacteria (*Bacillus anthracis*) in contaminated soil [12]. Its spores live naturally in the soil and are ingested by grazing animals such as cattle, sheep, goats and horses and wild herbivorous animals. The disease is of further importance because it presents a potential bioterrorism risk [13]. West and Central Africa including Benin, Burkina Faso, Ghana, the Niger and Togo have been frequently adversely affected by human and animal anthrax [12]. In animals, the disease is contracted from contaminated pasture and is almost always fatal. Previous studies from eastern Nigeria has reported prevalence rates of 5% and 3.3% in blood samples from cattle and goats slaughtered in an abattoir [14]. A more recent study carried out in a Nigerian abattoir recorded 10.5% sero-prevalence rate in livestock [15]. Food animals and their products constitute a potential risk to those persons involved in handling of animal and their products [14]. Human infection occurs via contact or consumption of infected dead animals or animal products [11]. Globally, there are about 2,000 to 20,000 human anthrax cases occurring annually [16]. About 425 human cases were reported in Nigeria between 1988 and 1995 [17, 18]. Depending on the route of exposure, clinical anthrax in humans presents in three distinct forms: cutaneous, gastrointestinal, and pneumonic form [19]. Vaccination using anthrax spore vaccine (Sterne attenuated spore vaccine), which is a non-pathogenic non-encapsulated variant strain of *B. anthracis* and is currently the effective method of prevention in animal population [20]. Effort are currently ongoing to develop an effective third generation vaccine for use in humans. Quarantine and containment to prevent movement of potentially infected animals or animal products, disposal of carcasses of affected animals by incineration and deep burial as well as use of disinfectant to treat contaminated soil [12].

**Bovine tuberculosis:** bovine tuberculosis (BTB), an infectious disease caused by *Mycobacterium bovis*. It is a slow growing, facultative intracellular, aerobic and gram-positive bacterium. *Mycobacterium bovis* a member of the *Mycobacterium tuberculosis* complex (MTC), which includes the closely related *M. tuberculosis*, the major causative agent of human tuberculosis. Bovine tuberculosis although a primarily cattle problem, it also infects other species of animals such as camels, pigs, sheep, goats, horses, dogs, cats, badgers, lions, elephants, deer, primates and man causing tuberculosis [21]. Bovine tuberculosis (BTB) is widespread and documented to be endemic yet poorly controlled in Nigeria based on report from several animal based studies [22-24]. Results from a past study by Cadmus, *et al* (1999) utilizing tuberculin skin test in selected cattle herds in some of the states of Nigeria revealed that the prevalence of up to 15.1% [25]. A detailed analysis of an abattoir record in Maiduguri, Nigeria, showed that 2,902 out of the 265,722 cattle slaughtered between May-June, 2008 were positive for pulmonary tuberculosis [26]. The WHO estimated that there were 147,000 new cases of zoonotic TB in humans and 12,500 deaths due to the disease globally [27]. Between 10 - 15% of human tuberculosis is considered to be caused by bovine tuberculosis [28]. Human infection occurs as a result of ingesting contaminated unpasteurized milk and raw meat and also by inhaling aerosol from infected livestock [29]. Human disease is characterized by the formation of granulomas (tubercles) in tissues and organs more significantly in lungs, lymph nodes, intestine, liver and kidneys [30]. Detection of bovine tuberculosis in Nigeria is carried out most commonly on the basis of tuberculin skin testing, abattoir meat inspection and rarely bacteriological techniques [31]. Antimicrobial treatment has been attempted in some species, but the treatment must be long term, and clinical improvement can occur without bacteriological cure. The risk of shedding organisms, hazards to humans and potential for drug resistance make treatment controversial. It is recommended that cattle with BTB should not be treated and as such farm animals with tuberculosis must be culled [32]. A human vaccine - *Bacillus* Calmette-Guérin (BCG) is widely available for prevention of the disease in humans of which *M. bovis* is the ancestor [33].

**Brucellosis:** brucellosis, caused by *Brucella species*, is considered an important neglected zoonosis by the WHO largely due to lack of public awareness and one of the most important endemic zoonotic infections, especially in pastoral and mixed crop-livestock farming systems in Africa [34-36]. It is the most common bacterial zoonosis affecting cattle, sheep, goats, pigs, and other animals worldwide, leading to reproductive problems such as abortion, infertility, and low milk yields, death from acute metritis, sterility, arthritis or bursitis (hygroma), and increased cost of animal replacement as well as lowered sale value of infected animals and loss in international trade [37]. There are also indirect economic losses for countries due to control and eradication of brucellosis [38]. Globally, about 500,000 new cases is reported annually, with prevalence rates in some countries exceeding 10 cases per 100,000 population [39]. Several studies have been carried out on bovine brucellosis in Nigeria with varying prevalence rates [40-42]. *Brucella* infections in humans can be contracted either directly or indirectly from exposure to infected animals and animal by-products or consumption of unpasteurized dairy products [43]. Brucellae organisms are shed in large numbers in the milk, urine, blood, and cystic products of infected animals [44]. Direct contact with animals or their secretions through cuts and abrasions in the skin, by way of infected aerosols inhaled or inoculated into the conjunctival sac of the eyes, or via the ingestion of unpasteurized infected milk and dairy products [45]. Symptoms of brucellosis in humans can be highly variable, ranging from non-specific, flu-like symptoms (acute form) to undulant fever, headache, malaise, night sweats, arthritis and arthralgia, etc [45]. Brucellosis also constitute an occupational risk for farmers, animal handlers, nomads, veterinarians, butchers, abattoir workers, animal products consumer and laboratory personnel [46,47]. A study carried out among abattoir workers in Nigeria revealed a very high sero-prevalence rate of 24.1% [48]. Incidence of human brucellosis with varying prevalence have also been reported in other parts of Africa including Tanzania [49]; Chad [50]. Calves should be vaccinated using the *Brucella abortus* strain 19 (S19) attenuated vaccine. Other management measures that involves proper disposal of aborted material, isolation and treatment of infected animals and routine testing will reduce burden of the disease in animal population thus prevents human infection. Human at high risk such as abattoir workers should be use personal protective equipment while handling meat products.

**Cysticercosis (taeniasis):** this is a zoonotic helminth infection caused by tapeworm *Taenia solium* and is widespread in Africa. Cysticercosis imposes substantial global burden on human beings related to epilepsy, ocular disorders, other neurological manifestations, and economic losses related to disability and lost productivity [51]. A study by Biu and Ijudai (2012) in pigs reported a porcine cysticercosis prevalence of 9.5% in Northern Nigeria [52]. However, a higher prevalence of porcine cysticercosis based on a detailed abattoir examination of pig carcases was observed in another study carried out in Nigeria with up to 20.5% of pigs infected [53]. Human cysticercosis is acquired from the ingestion of food contaminated with ova of *T. solium*, with subsequent development of cyst in human tissue [54]. An estimated 2.5 million people are infected with *T. solium*, and there are approximately 50,000 deaths annually due to neurocysticercosis [54]. Neurocysticercosis is a complication of infection with *T. solium*and is considered to be the most common preventable cause of acquired epilepsy in the developing world accounting for about 30% of epilepsy in humans, and the most important neurological disease of parasitic origin in humans [11]. In sub-Saharan Africa, people with cysticercosis have been estimated to have a 3.4. to 3.8-fold increased risk for developing epilepsy [55]. Furthermore, Zoli, *et al* (2003), estimates that annual economic losses due to porcine cysticercosis in 10 West and central African countries amount to about 25 million Euros of which 2 million attributed to Nigeria [56]. In 2012, epilepsy associated with neurocysticercosis was responsible for an estimated 17,853 incident cases and 212 deaths in the United Republic of Tanzania [57]. Human cysticercosis has been reported with an estimated prevalence rate of up to 8.6% in hospital patients in Nigeria [53]. In another study carried out by Weka *et a*l (2013), 9.6% of pig rearers tested in Jos, Nigeria had positive antibodies to *T. solium* using IgG antibody ELISA technique [58]. A recent study carried out among school children in Southern Nigeria, revealed a very high prevalence rate of about 40.9% by faecal examination [59].

**Echinococcosis (hydatidosis):** hydatidosis is a neglected parasitic disease of public health importance caused by the larval stage of cestode of the genus Echinococcus [60, 61]. *Echinococcus granulosus* and *E. multilocularis* are species of major public health importance and are responsible for virtually all the human and animal burden of the disease causing human cystic echinococcosis (CE) and alveolar echinococcosis (AE), respectively [62, 63]. The life cycle of the tapeworm (*E. multilocularis*) is sustained between the definitive hosts which are domestic dogs, which exhibit canine echinococcosis and the intermediate hosts for the larval stages (metacestodes) such as sheep, goats, cattle, pigs, equines and camelids (herbivorous animals) in which cystic hydatid disease occurs [60, 63]. The disease results in serious economic loss to the livestock industry through condemnation of infected organs in food animals [63]. A very high prevalence rate of 56% in pigs, 42% in goats, 32% in cattle and 24% in sheep was reported in past studies in Nigeria [64]. A recent study utilizing serology reported 12.45% prevalence rate in dogs [60]. Humans are accidental hosts. They become infected with the disease via accidental intake of parasitic eggs excreted by the faeces of definitive hosts such as dogs, foxes and other wild canids such as African wild dogs, wolves and jackals [63]. Eating infected offal by humans and animals is also one of the important risk factor of infection for canine echinococcosis [63, 65]. They also get infected through the consumption of contaminated raw vegetables or from handling infected dogs. Humans develop cystic lesions, principally in liver and lungs, and other organ systems [61, 66]. An annual loss of about US$193,529,740 has been estimated for human echinococcosis while loss to the global livestock industry has been estimated at over US$2 billion annually [61]. It has been reported to be one of the costliest diseases to treat and prevent in terms of public health [67].

**Human African trypanosomiasis:** human African trypanosomiasis HAT, also known as sleeping sickness, is a vector-borne parasitic disease caused by infection with protozoan parasites of the genus *Trypanosoma*. Trypanosoma is transmitted through bites by different tsetse fly of the genus *Glossina*. Trypanosomes are pathogenic to animals and cause animal trypanosomiasis in wild and domestic animals. Wild and domestic animals are an important reservoir for HAT. Human African Trypanosomiasis in endemic countries of sub-Saharan Africa with an estimated 65 million people are at risk of the disease and between 50,000, 70,000 cases and about 17,000 new cases annually [68]. In Nigeria, about 100 new cases are reported annually [69]. A review of mortality rate due to HAT on data available from Nigeria between 1970-1979 showed a total mortality of 0.003% out of approximately 28,000 infected persons with the highest deaths recorded in 1974 (18.1%) and 1972 (17.7%) [70]. Human African trypanosomiasis takes 2 forms, depending on the parasite: Those caused by *Trypanosoma brucei gambiense* which accounts for 98% of sleeping sickness and found mostly in Western and Central Africa. The second form is caused by *Trypanosoma brucei rhodesiense* accounting for the rest of the human cases and found in Eastern and Southern Africa [71]. The disease has been recently re-emerging in parts of Africa including Nigeria. A prevalence study utilizing card agglutination test for trypanosomiasis kit detected 9.6% of human subjected tested were positive in an endemic area of Nigeria [72]. In a recent case, HAT caused by *Trypanosoma brucei gambiense* acquired in Nigeria and imported into the United Kingdom has been reported [73].

**Leishmaniasis:** this neglected, protozoan parasitic disease is considered to be the third most important vector-borne disease worldwide. It is a zoonotic disease with an estimated 350 million people are at risk in 88 countries, 12 million human cases reported worldwide, and about 50,000 deaths annually [74]. Domestic and wild dogs, small rodents are the most important animal reservoir hosts of leishmaniasis, which is transmitted among canines and to humans by the female *Phlebotomus* sand flies belonging to insect order Diptera - true flies [75]. *Leishmania* parasites cause three forms of leishmaniases according to the localization of the parasites in mammalian tissues, notably visceral, cutaneous, and mucosal leishmaniasis [76]. *Leishmania infantum* is the aetiological agent of canine visceral leishmaniasis [77]. The disease is mostly chronic in dogs with clinical signs including anemia, hyper-gammaglobulinaemia, generalized lymphadenopathy, skin lesions, epistaxis, and chronic renal failure and eventual death [75]. Dogs with clinical disease presents mainly ulceration, alopecia and desquamation of skin and generalized nodular lesions or pustules [78]. In endemic area, dogs that recover after treatment from infection or those that are asymptomatic continue to be reservoirs of infection, hence they should be euthanized to prevent spread of the disease [75]. In a recent study carried out in Kwara State, Nigeria, a sero-prevalence rate of 14.63 % was reported for canine leishmaniasis [79]. The first case of human cutaneous leishmaniasis was reported in Nigeria in 1924 [80]; and recently human cases are still being reported [81, 82]. Clinical lesions of cutaneous leismaniasis (CL) include skin ulceration, multiple nodular swelling [82]. Visceral leishmaniasis is the severe form of the disease leading to 100% mortality if left untreated [83]. Between 1992 and 1995, two patients suffering from CL died in Nigeria due to lack of timely access to antimonials [84].

**Rabies:** rabies remains a major concern globally [85]. It is a fatal zoonotic disease caused by a rabies virus of the genus Lyssavirus, family Rhabdoviridae and order Mononegavirales [86]. Humans get infected following bites of an infected animal or contact with saliva of an infected animal from where it migrates to the brain. It has been classified as the eleventh killer disease of the world, killing 60 - 100, 000 human beings annually mainly in Africa and Asia. It is recommended that in order to prevent rabies in endemic regions, effective and efficient vaccination of a minimum of 70% of the dog population in a target area should be carried out [87]. Mass vaccination of the animal reservoirs is the cost-effective method of prevention as because post-exposure prophylaxis in humans is much costlier [88]. The cost of rabies is substantial as according to the WHO, about 15 million people received post-exposure prophylaxis annually [89]. Rabies is well known by the indigenous people based on the availability of various indigenous terms - that the disease had been recognised in Nigeria and known as: *ciwon kare (Hausa), ara nikita (Igbo) and digbolugi (Yoruba)* [90]. However, the first reported case of rabies in animals was in 1925 while the first human case was in 1912 in Nigeria [91]. Since then, canine rabies is an endemic and widespread from various parts of Nigeria [92]. Six people out of 149 suspected dog bite cases were confirmed to have human rabies in a study carried out recently in Nigeria [93].

**Role of the public health veterinarian in neglected zoonotic disease control:** WHO defines veterinary public health as, “The sum of all contributions to the complete physical, mental and social well-being of humans through an understanding and application of veterinary medical science” [94]. Veterinary public-health activities are those activities conducted at the human?animal interface; that are required to prevent, control and eliminate suffering and economic loss caused by NZDs in both humans and animals [1]. Veterinary public health is devoted to the application of veterinary skills and knowledge for the protection and improvement of public health [95]. Zoonotic diseases such as elucidated above are not wholly veterinary medical problem but also serious public health concern, therefore rapid detection and control in animal population is important for control of human zoonotic infection. The importance of utilizing One Health approach to successfully manage and control zoonotic diseases has been emphasized [5, 11, 96]. Thus effective preventive measure should involve a “One Health” multidisciplinary approach such that the disease can be controlled in both human, animal and the environment. There are several important roles that the Veterinarian is engaged in the area of preventing NZDs and a few but not exhaustive are as follows:

**Response to emerging and re-emerging zoonotic diseases:** according to the WHO, the expertise of a public health veterinarian is essential in the public health infrastructure for efficient preparedness and response to known and unexpected disease problems [94]. Since several neglected zoonotic diseases have animals as either vectors, intermediate hosts or directly transmit the pathogens to man, involvement of the public health Veterinarian in a multidisciplinary emerging zoonotic disease outbreak response, would ensure that these diseases are controlled at the source. They are able to prevent disease from occurring in the human population by controlling the vectors and vehicles such as aborted foetuses in brucellosis outbreak, control of NZDs in wildlife reservoirs. Field veterinary epidemiologists are also involved in ambulatory services to investigate reports of suspected outbreaks of NZDs. This type of response is able to nip an outbreak in the bud and appropriate measures such as test and slaughter, culling, ring vaccination etc are carried out.

**Neglected zoonotic disease surveillance:** the veterinary epidemiologist and public health specialist are involved in disease surveillance in animal population and wildlife. A public health Veterinarian should be involved in joint disease reporting from clinical and veterinary diagnostic laboratories as a part of public health surveillance. They monitor trends in disease occurrence in the abattoir, clinics and research centres. Neglected zoonotic diseases such as BTB, hydatidoses, taeniasis can be easily detected in the abattoir. Both ante and post mortem inspection of animals in the abattoir plays a key role in both the surveillance network for animal diseases and zoonoses and ensuring the safety and suitability of meat and by-products [97]. Suspected or confirmed cases are condemned and only wholesome meat and meat products are sent to the human population. This considerably stops the spill over of infection from the animal to human population. Although, in Nigeria, there are cases that might be missed due to lack of adequate meat inspection infrastructure, animals that are brought into government owned abattoir are inspected and certified by the Veterinarian.

**Prevention of animal diseases:** for NZDs that are vaccine preventable such as rabies, routine vaccination schedule is adopted and dogs are vaccinated. Vaccinated dogs are not only protected from the deadly disease, but reduces considerably the possibility of human rabies as over 90% of human deaths from rabies worldwide are caused by dog bites [98]. Prevention of human rabies is dependent on eliminating dog rabies. Mass rabies vaccination of dogs has been shown to be effective in developed countries of the world. The public health Veterinarian is also involved in other components of zoonotic diseases preventive measures such as brucellosis vaccination campaign, prompt and immediate treatment of animal diseases including those that are of public health significance. Others are in the areas of vector control and environmental health.

**Development of vaccines for neglected zoonotic diseases:** veterinary vaccines are important cost-effective method for protecting animal health and prevent transmission of zoonotic and foodborne infections to the public [99]. There are Veterinarians specialized pharmacology, toxicology, microbiology and are involved in the production of drugs, vaccines and biological products for human and animal use. Vaccines for NZDs such as those against rabies, brucellosis was effectively utilized to control the diseases in most developed countries due to availability of effective vaccines [99].

**Research, policies and zoonotic disease control guidelines:** with recent advances in research such as geographic information systems (GIS), molecular epidemiology, evolutionary and transmission dynamics studies, the public health Veterinarian is involved in extensive studies to understand the linkages between human and zoonotic diseases [94]. Some of these include identification of zoonotic disease hotspots, modelling and prediction of location and population at risk. The public health Veterinarian is also involved in policy and governance and is able to develop policies and implementation guidelines towards a sustainable control of neglected zoonotic disease. A lot of NZDs that were eradicated in the developed nations were as a result of good policy formulation and implementation. Proper legislation and administration as well as the implementation of disease control programs would be beneficial such as meat inspection laws, veterinary drug control etc. Although a lot is still left to be desired in developing countries including Nigeria in implementation of these laws, the importance of the Veterinarian in ultimately protecting human health through healthy animals is well established.

## Conclusion

The majority of people from Nigeria are dependent on livestock for their livelihood. This means of livelihood allows close interaction of human with their animals and even contact with wild animal due to nomadic nature of pastoralists and farmers. There is potential exchange of infection between man and animals, some of which belong to the “neglected category”. There is a very important role for the Veterinarian to control the NZDs from animal reservoir and involvement in policy issues related to zoonotic diseases control in order to prevent devastating human outbreaks. The link between human and veterinary medical practitioners in Nigeria is weak. There has been recent drive to improve multidisciplinary collaboration such as the establishment in 2011, the Nigeria Centre for Disease Control (http://ncdc.gov.ng/). There should be improved areas of collaboration such as coordinated surveillance to intensively monitor any emergence or change in the disease occurrence both within the animal and human population as well as population most at risk. Additionally, sharing of disease event data between health and veterinary ministries and department is likely to improve synergy in NZDs control. A previous review article on control of zoonotic disease in Nigeria similarly suggests partnership between animal and human medical practitioners [100]. A sustainable surveillance program involving veterinary and medical experts both nationally and internationally for a successful zoonotic disease control program is therefore emphasized. Furthermore, it was noted from the review of literature, that up to date nationwide baseline survey is lacking in Nigeria to determine the current status of human NZDs burden. It is therefore suggested that a largescale research to determine the current burden of human neglected zoonotic diseases should be commissioned. Indeed, one of the reason for neglect is the absence of data on the burden of these diseases in the country, to warrant instituting timely and sustainable control measures. These research should be multidisciplinary and should include all the geopolitical zones of the country. This should also utilize extensive review of hospital and other surveillance record for retrospective pattern of disease occurrence. Also a broad range of new generation methods that will ensure that reliable data are generated for control should be utilized for prospective studies.

### What is known about this topic

- We know that a sub-set of tropical diseases are neglected zoonoses and affect rural people that come in contact with animals mostly in sub-Saharan African;
- We know that these diseases have been documented in the Nigeria among both people and animal population.

### What this study adds

- This review provides a single document bringing together the neglected zoonotic diseases so far documented in Nigeria from literature search;
- It also provides a comprehensive detail of the current status of these NZDs with relation to burden and public health impact among Nigerian human and animal population;
- It documents as a reference, the very important role of Veterinarians in controlling these NZDs and made important recommendations to strengthen the role of the public health Veterinarian.

## Competing interests

The authors declare no competing interests.
